# *Strongyloides stercoralis* Infection in Alcoholic Patients

**DOI:** 10.1155/2016/4872473

**Published:** 2016-12-26

**Authors:** Marcia C. A. Teixeira, Flavia T. F. Pacheco, Joelma N. Souza, Mônica L. S. Silva, Elizabete J. Inês, Neci M. Soares

**Affiliations:** Faculdade de Farmácia, Universidade Federal da Bahia, 40170115 Salvador, BA, Brazil

## Abstract

The course of* Strongyloides stercoralis* infection is usually asymptomatic with a low discharge of rhabditoid larva in feces. However, the deleterious effects of alcohol consumption seem to enhance the susceptibility to infection, as shown by a fivefold higher strongyloidiasis frequency in alcoholics than in nonalcoholics. Moreover, the association between* S. stercoralis* infection and alcoholism presents a risk for hyperinfection and severe strongyloidiasis. There are several possible mechanisms for the disruption of the host-parasite equilibrium in ethanol-addicted patients with chronic strongyloidiasis. One explanation is that chronic ethanol intake stimulates the hypothalamic-pituitary-adrenal (HPA) axis to produce excessive levels of endogenous cortisol, which in turn can lead to a deficiency in type 2 T helper cells (Th2) protective response, and also to mimic the parasite hormone ecdysone, which promotes the transformation of rhabditiform larvae to filariform larvae, leading to autoinfection. Therefore, when untreated, alcoholic patients are continuously infected by this autoinfection mechanism. Thus, the early diagnosis of strongyloidiasis and treatment can prevent serious forms of hyperinfection in ethanol abusers.

## 1. Introduction

*Strongyloides stercoralis* infection is prevalent in countries with tropical and subtropical climates and affects approximately 370 million people worldwide [1, 2]. The helminth has the ability to multiply within a host, regardless of the mode of exogenous contamination, due to the transformation of rhabditoid larvae into infective filariform larvae, leading to persistent infections. However, in most hosts, the course of parasitism remains quiescent with no significant morbidity. This parasite-host balance can be disrupted under conditions of impaired cellular immunity, resulting in the life-threatening strongyloidiasis condition [3]. Immunosuppressed patients with chronic strongyloidiasis are at high risk of developing serious complications, such as hyperinfection syndrome and the dissemination of the parasite to several organs, causing sepsis and even death. High risk groups for* S. stercoralis* infection and hyperinfection include patients under massive corticoid therapy, HTLV-1 coinfected individuals, and chronic alcoholics [4–6].

Many studies have demonstrated that chronic alcohol abuse predisposes an individual to* S. stercoralis* infection. In alcoholics with hepatic cirrhosis, the infection may evolve to hyperinfection and life-threatening strongyloidiasis. The high predisposition to* S. stercoralis* infection has been associated with poor hygiene practices, malnutrition, the impairment of protective immune responses induced by excessive alcohol intake, and increase in endogenous corticoid levels, favoring* S. stercoralis* autoinfection [5, 7].

Correct diagnoses, treatments, and clinical follow-ups of parasitological cures are essential because strongyloidiasis relapse events have often been observed in patients receiving improper treatment [8]. Despite the high prevalence of strongyloidiasis in endemic countries and an increasing number of fatal cases, the World Health Organization (WHO) did not include strongyloidiasis in its original list of 17 neglected tropical diseases. Therefore, the main purpose of this article is to review the association between strongyloidiasis and chronic alcoholism, including describing the organic changes induced by both pathologies; the adverse effects of alcohol that predispose an individual to* S. stercoralis* infection and hyperinfection; and the current diagnostic tools and therapies for strongyloidiasis.

## 2. *Strongyloides stercoralis* Infection and Strongyloidiasis

The* Strongyloides stercoralis* threadworm is a soil-transmitted nematode that resides in the small intestine of human hosts. The parasitic infection takes place when filariform larvae penetrate through the skin, usually of the feet, and migrate through the bloodstream to the lungs [9]. After ascending the respiratory tract to the oropharynx, larvae are swallowed and reach the duodenal mucosal crypts to grow into parthenogenetic females that produce embryonated-eggs. Thereafter, rhabditoid larvae hatch from the eggs and are excreted in feces. However, some larvae may transform into the filariform infective stage and penetrate the perirectal mucosa or skin, thereby reentering the circulatory system and starting the cycle again. Therefore, if not treated, because this is an autoinfection process, the host may remain in a chronic carrier state for decades [10].

*Strongyloides stercoralis* usually causes an asymptomatic infection and a small rhabditoid larvae load in feces [11]. However, hyperinfection and dissemination can occur in high risk groups, such as patients undergoing glucocorticoid therapy [12], patients coinfected with HTLV-1 [13] or HIV [14], lymphoma patients [15], and people with malnutrition [16] or with liver cirrhosis due to alcoholism [17].

The clinical manifestations of infection with* S. stercoralis* comprise a broad spectrum of signs and symptoms and can be divided into four clinical presentations: (a) acute strongyloidiasis, (b) chronic strongyloidiasis, (c) hyperinfection syndrome, and (d) disseminated disease. The clinical signs of acute strongyloidiasis are associated with the penetration of larva in the host and their passage through the lungs. Infected individuals may experience an itchy papular rash at the site of invasion. Depending on the number of larvae, their passage through the lungs can produce bronchospasm, coughing, and respiratory distress stemming from eosinophilic pneumonitis and leading to Loeffler syndrome, which is characterized by pulmonary infiltrations in chest radiographies and peripheral blood eosinophilia. When parthenogenetic females reach and colonize the intestinal mucosa, recurrent abdominal pain may occur sometimes resembling peptic ulcers [6, 18].

Chronic strongyloidiasis most frequently leads to an asymptomatic infection in immunocompetent individuals. Peripheral eosinophilia or elevated total IgE levels greater than 250 IU/mL may be observed in up to 75% of infected hosts [19], serving as a marker for differential diagnoses for travelers to or immigrants from endemic areas with high and persistent levels of eosinophilia [20]. In symptomatic patients with chronic diseases, nonspecific gastrointestinal manifestations, such as abdominal pain, vomiting, diarrhea, and constipation, may also be observed [11].

*S. stercoralis* hyperinfection is considered to be an enhanced form of autoinfection and is typically, but not always, a result of changes in the immune status [21]. In hyperinfection syndrome, signs and symptoms of increased larval migration appear:* Larva Currens* in the perianal region, recurrent asthma, and exacerbation of gastrointestinal symptoms, followed by the detection of large numbers of larvae in sputum and feces [22, 23]. Radiographs frequently demonstrate focal or bilateral interstitial infiltrates in lungs. A retrospective study analyzed the endoscopic findings of 25 patients, mostly being treated for HTLV-1 or under corticosteroid therapy. These patients presented with* S. stercoralis* hyperinfection before beginning parasitological treatments for strongyloidiasis and revealed mostly edematous and erythematous mucosa, intestinal mucosa erosion, white villi, stenosis, and hemorrhage [24]. Duodenitis, including villous atrophy and destruction and inflammatory cell infiltration, was more severe in patients whose duodenal biopsies were positive for larvae than in those with negative biopsies [24]. As described above, hyperinfection syndrome implies, as interpreted by many clinicians, the presence of signs and symptoms attributable to increased number of migrating larvae through intestinal tract and lungs. However, some patients may continuously excrete huge parasite loads in feces and remain asymptomatic, likely due to the protection afforded by high levels of specific IgE [25]. Independent of these symptoms, in hyperinfection, the larvae are located in organs which normally carry the infection cycle without spreading to ectopic locations [6, 22].

Disseminated infection indicates the migration of the larvae to organs away from the normal cycle path inside a host (i.e., the lungs and the intestine). Infective larvae can be found virtually in any organ or system, such as the central nervous system, lymph nodes, heart, pancreas, kidneys, ovaries, and skeletal muscles, as indicated by clinical manifestations [26]. Large numbers of larvae penetrating through the intestinal wall may carry bacteria into the bloodstream and result in systemic infections, which may occur even during hyperinfection. The disseminated strongyloidiasis is often fatal, because of its rapid evolution and late diagnoses [3].

## 3. Epidemiology of Strongyloidiasis and Association with Alcoholism

Strongyloidiasis has a wide geographic distribution especially in populations of developing countries of tropical and subtropical areas. Various factors can influence* S. stercoralis* prevalence, including the integrity of immunity, socioeconomic status, sanitary and hygiene conditions of individuals, the level of heat and humidity, which affect parasite development in soil [27, 28], and the laboratory methods used for diagnosis [29, 30]. The prevalence of strongyloidiasis in a population can be divided into three categories: sporadic (<1%), endemic (1–5%), and hyperendemic (>5%) [31].

According to a systematic review based on published* Strongyloides* infection rates and taking into account the sensitivity of the used diagnostic methods, the prevalence of* Strongyloides* in Africa varied from 0.1% in the Central African Republic to up to 91.8% in Gabon. In South America and Central America, Haiti reported a prevalence of 1.0%, while in Peru the infection rate was as high as 75.3%. In Cambodia, the infection rate was 17.5%, whereas in Thailand, the rate was 23.7% and in Lao PDR, the rate was 26.2% [29]. Brazil is considered a hyperendemic country with 5.5% of* S. stercoralis* infection in general population and 11.8% in immunosuppressed individuals, considering the parasitological diagnosis [32]. Moreover, there are areas of low endemicity in western European countries (France, Italy, and Switzerland), Eastern Europe (Poland and areas of the former Soviet Union), the United States (Appalachia and southern states), Japan (Okinawa), and Australia (Aboriginal people) [29, 33, 34].

In patients who chronically use alcohol, the prevalence of infection by* S. stercoralis* is usually high, ranging from 20.5% to 40.2% [5, 7, 17, 35]. A study conducted in Brazil showed a 33.3% infection frequency in alcoholic patients. This value was even higher (44.4%) in patients with liver cirrhosis, whereas only 5.5% of nonalcoholic individuals were infected [35]. The amount of alcohol intake was proportional to the increased infection frequency, reaching 48% in patients who drank more than 450 g of alcohol per day [5]. Moreover, 40.2% of patients with liver cirrhosis of alcoholic etiology treated in a university hospital of Brazil had* S. stercoralis* infection [17]. Another interesting study conducted in Brazil showed that, in a group of 100 patients with HIV/AIDS, 12% were infected with* S. stercoralis* and of these, 64.3% were chronic alcoholics [14], suggesting a synergism between the susceptibility factors. In addition, a recent meta-analysis of three case-control studies showed an association between alcoholism and* S. stercoralis* infection with a significant increase in the risk of infection in alcoholics (OR: 6.69; CI: 1.47 to 33.8) [29]. Conversely, a study in Costa Rica found a lower rate of 5.7% (6/106) of* S. stercoralis* infection in alcoholic patients when compared with the frequencies found in Brazil. However, the rate of infection in the entire Costa Rica population was 0.1%, indicating that alcoholics were four to five times more susceptible to* S. stercoralis* infection [36], as observed in Brazilian studies. In fact, our group reported an occurrence of 23.5% of* S. stercoralis* infection in alcoholics from the Brazilian Northeast [37].

There are also case reports that have shown the association between alcoholism and* S. stercoralis* infection. A patient attended by our laboratory without gastrointestinal or pulmonary symptoms presented with very intense anemia and a high discharge of* Strongyloides* larvae in his stool. He was not under glucocorticoid therapy and tested negative for HTLV and HIV but had a history of alcohol addiction for more than 20 years [25]. Another case described a colitis caused by* S. stercoralis* in a woman dependent on alcohol and without the HTLV-1 virus. The patient had been infected for 27 years with worsening chronic strongyloidiasis due in part to the impairment of her cellular immune response resulting from the chronic use of alcohol and malnourishment [38].

In immunocompromised patients, including alcohol addicts, chronic strongyloidiasis may persist for decades. Additionally, due to the autoinfection mechanism of the parasite, chronic strongyloidiasis may result in hyperinfection and spread to other organs [6, 18, 25]. Despite the fact that alcoholism is one of the conditions observed in patients with* S. stercoralis* hyperinfection, only in the past decade controlled studies showed that strongyloidiasis is more frequent in alcoholic than nonalcoholic patients [7, 35].

**Figure 1:**
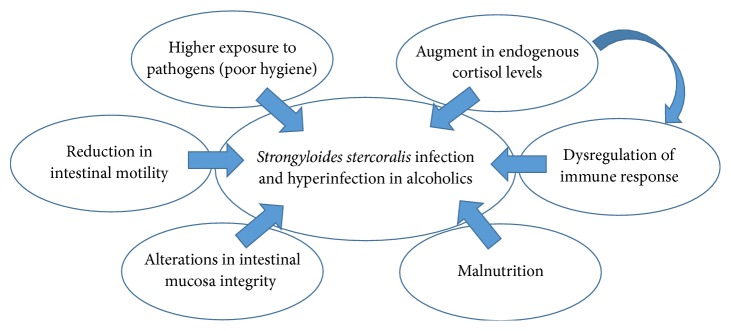
Factors associated with increased* Strongyloides stercoralis* infection and hyperinfection susceptibility in alcoholics.

Of the factors contributing to the high susceptibility to* S. stercoralis* infection, the poor hygiene of alcoholics promotes heteroinfection in environments with inadequate sanitary conditions and autoinfection by larvae present in fecal residue on the perianal skin [5]. Another factor that contributes to high infection rates could be attributed to reductions in gastrointestinal transit, which are caused by the effects of ethanol on intestinal muscle proteins or on vagal stimulation [39, 40]. Because the reduced intestinal motility enables the rhabditoid larvae to mature to the filariform infective stage, the risk of autoinfection is increased. Other authors have also emphasized that the dysregulation of the immune system caused by excessive ethanol intake may promote the survival of the parasite and autoinfection by increasing the endogenous production of corticosteroids [7, 35]. Ethanol intoxication has been shown to activate the HPAaxis, which elevates corticosteroids levels [41–44], which in turn suppresses T-cell function and decreases intestinal immunity [45, 46]. Alcoholics may also present reduced numbers of macrophages in the duodenal mucosa and deficiencies in IgA secretion, which could be partially responsible for the high incidence of* S. stercoralis* intestinal infections [47, 48]. A schematic figure with the suggested mechanisms for higher predispositions of ethanol-addicted individuals for strongyloidiasis is presented in Figure 1.

## 4. Immune Response to* S. stercoralis* and Immunity Alterations Induced by Chronic Alcohol Intake

As in most helminth infections, the dominant cellular immune response to* S. stercoralis* is Th2. The IL-4 and IL-5 interleukins stimulate the production of IgE, which in turn induces mast cell degranulation and mucus secretion by goblet cells [49]. Peristalsis induced by IL-4 and IL-13 with the mucus facilitates the expulsion of the helminths, while toxic granules released by mast cells can cause direct damage to the parasite [50]. Moreover, IL-4 and IL-5 promote the activation of eosinophils, which plays an important role in host defense. Eosinophils are not only directly involved in the innate immune response against helminth larvae but are also involved in the adaptive immune response [49, 51, 52]. They act as antigen-presenting cells and increase the production of Th2 cytokines, such as IL-4, IL-5, and IL-13, and, consequently, the production of specific antibodies IgE, IgG, and IgM that eliminate the parasite [51, 53, 54]. Furthermore, the degranulation of eosinophils on the surface of the parasite through antibody-dependent cellular cytotoxicity (ADCC) results in the release of toxic molecules that induce the removal of helminth [55].

In patients infected with* S. stercoralis*, eosinophilia can be more frequent than in other parasite infections. This phenomenon is caused by the habitation of parthenogenetic females in the intestinal submucosa and not in the lumen, which results in a more intense eosinophilic reaction [2, 56]. The eosinophil-dependent mechanisms are also involved in filarial larvae killing of S*trongyloides* [57, 58]. Therefore, the absence of eosinophils is a poor prognostic indicator for infection by* S. stercoralis*, particularly for immunocompromised patients [59].

The role of antibodies in protective immune responses against* S. stercoralis* has been demonstrated in the passive transfer of anti-*S. stercoralis* antibodies to naïve animals [60]. Furthermore, the humoral immune response plays an important role in autoinfection control because IgA antibodies limit the amount of secreted larvae presumably by inhibiting the fecundity of the parthenogenetic female and the viability of eggs. The binding of IgE to effector cell receptors, especially those of mast cells and basophils, induces degranulation and the release of inflammatory mediators, which lead to the death and expulsion of the worm. In addition, the binding of IgE to mast cells present in the intestine releases sulfated proteoglycans, which not only hamper the establishment of* S. stercoralis* in the intestinal epithelium but also stimulate muscle contraction. These mechanisms contribute to the expulsion of the intestinal parasite [58, 61, 62]. Conversely, IgG4 can block the IgE-mediated immune response, contributing to the persistence of asymptomatic strongyloidiasis [63].* Strongyloides* infection can also stimulate regulatory T cells (T reg), an escape mechanism of the parasite, which suppresses the protective immune response, such as the eosinophil-dependent activation by IL-5 [64].

Current medical literature recognizes that excessive alcohol use is associated with reduced host defense, including antimicrobial defense, antiviral immunity, and altered host repair [65–67]. Alcohol leads to the stimulation of the HPA axis and produces excessive levels of cortisol [68]. There is high correlation between the neuroendocrine and immune systems, especially in the sensitivity of the immune system to stress and its interaction with the HPA axis. This interaction was principally revealed by the immunosuppressive actions of glucocorticoids, especially cortisol, which can affect the transcription of numerous inflammatory molecules [68]. This interaction between the HPA axis and the immune system is crucial for body homeostasis; however, in alcohol abuse conditions, the equilibrium of this interaction is compromised. At excessive quantities, glucocorticoids have serious adverse effects due to their immunosuppressive action and metabolic abnormalities [69, 70]. Moreover, glucocorticoids upregulate IL-18, which enhances myeloperoxidase activity and alters the intestinal barrier function [71]. Neutrophils may also play a role in increasing intestinal permeability in both acute and chronic ethanol intoxication [45, 72]. The IL-18-mediated increase in chemokines and adhesion molecules likely causes intense neutrophil accumulation in the intestinal tissue, contributing to the impairment of intestinal immunity [45, 71].

**Figure 2:**
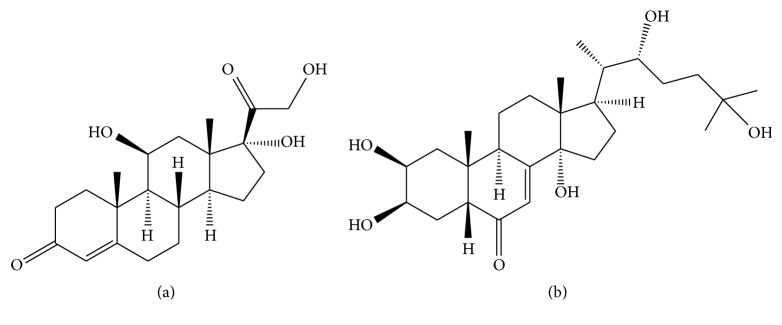
Chemical structure of cortisol (a) and ecdysone (b). Adapted from Wikipedia.

The strong association between high alcohol use and heightened cortisol levels also indirectly contributes to* S. stercoralis* autoinfection. Hydrocortisone, or cortisol, is a corticosteroid hormone produced by the cortex of adrenal glands directly involved in stress response. Corticosteroids produce metabolites that resemble hydroxyecdysone (Figure 2), an ecdysteroid hormone that regulates the fertility of parthenogenetic* S. stercoralis* females, induces the transformation of rhabditiform to infective filariform larvae, and increases the rate of autoinfection [8, 18, 25]. The presence of a steroid receptor on* S. stercoralis* could be involved in the pathogenesis of hyperinfection syndrome and disseminated strongyloidiasis [18, 73]. The cortisol metabolites could bind to this receptor and exacerbate the transformation of rhabditoid larvae to the filarial stage. This action leads to the invasion of the intestinal mucosa, resembling the autoinfection mechanism observed in patients under massive corticosteroid therapy [73, 74]. In fact, we have recently observed that high endogenous cortisol levels in alcoholic patients may not be associated with susceptibility to* S. stercoralis* infection; however, once infected, this may lead to a high parasite load [37].

In contrast to the inhibitory effects of acute alcohol ingestion, prolonged alcohol intake results in increased macrophage TNF-*α* production and the activation of the inflammatory cascade [75, 76]. In alcoholic hepatitis, a disease resulting from chronic alcohol intake, the levels of proinflammatory cytokines, such as TNF-*α*, IL-1, and IL-6, are highly elevated [77]. The highest TNF-*α* levels were correlated with liver dysfunction. However, light-to-moderate drinking had no significant effect on the levels of serum TNF-*α* [78].

There are two proposed mechanisms by which chronic alcohol use can induce inflammation: (1) gut microflora-derived lipopolysaccharides (LPS), which act as key players in alcohol-mediated inflammation [79] and alcohol metabolism through the production of reactive oxygen species (ROS), and (2) cell damage, which initiates the production of proinflammatory cytokines, such as TNF-*α* and IL-6 [80]. Regarding cytokine production, one possible mechanism is an increase in the TLR4 stimulation-induced proinflammatory cytokine production and NF-kB activation [75]. However, during the overactivation of monocytes, such as during the combined stimulation of TLR2 and TLR4, proinflammatory cytokine production can be increased even with acute alcohol ingestion [75]. A few studies have demonstrated that both acute and chronic alcohol consumption enhanced the expression of anti-inflammatory cytokines [76, 81].

Acute and chronic alcohol exposure can interfere with several aspects of the adaptive immune response [82]. Acute alcohol intoxication impairs the antigen-presenting ability and differentiation of dendritic cells (DC). This effect is more potent in affecting antigen presentation and antigen-specific T-cell activation than in monocytes or macrophages [83]. The alcohol-induced defects in DC functions include reduced levels of CD80 and CD86 on the cell surfaces (essential to induce activation of T cells) and decreased production of IL-12 (critical for stimulating naïve CD4^+^ T cells to develop into Th1 cells) [82]. Thus, acute alcohol intake may inhibit the Th1 immune response and may predispose the host organism to Th2 responses. The suppression of IL-12 seems to be partially responsible for this shift [82]. The activation of Th2 as an anti-inflammatory response seems to coincide with the cytokine expression profiles observed during acute alcohol intake. It is expected that during chronic alcohol use, a Th1 response dominates over a Th2 response. Therefore, the hyperinfection of* S. stercoralis* in chronic alcoholics could be explained by the same mechanism observed in the* S. stercoralis*-HTLV-1 coinfection. The shift from Th2 to Th1 immune responses resulted in decreased levels of IL-4, IL-5, IL-13, and IgE and the synergistic severity of the disease [84]. Another possible mechanism for the impaired Th2 response in alcoholics may be associated with Th2 cell apoptosis induced by increased endogenous glucocorticoid levels, leading to* S. stercoralis* hyperinfection [74]. However, it was demonstrated that chronic alcoholics have elevated levels of IgE, a typical immunoglobulin involved in Th2 response [85]. Therefore, further studies are needed to clarify the mechanisms of immune response in alcoholic individuals coinfected with* S. stercoralis.*

## 5. *S. stercoralis* Diagnosis and Treatment

The clinical diagnosis of strongyloidiasis is presumptive because the signs and symptoms are nonspecific and can be confused with those of other intestinal parasitosis [86]. Currently, laboratory diagnoses are routinely performed to identify rhabditoid larvae in feces. However, in most cases, the parasite load is low and the shedding of larvae is intermittent, which can interfere with the efficiency of parasitological methods [87, 88]. Therefore, to increase the sensitivity of parasitological examination, at least three stool samples must be analyzed on alternate days using different diagnostic methods [89–91]. However, the repeated delivery of samples to the laboratory may be inconvenient for patients due to distances between residences and the laboratory and extended travel times. Because of these factors, a sufficient number of samples are not always examined.

Several parasitological methods are used for the detection of* S. stercoralis* larvae in feces, such as the Baermann-Moraes [92], Harada-Mori filter paper culture [93], agar plate culture (CPA) [94], TF-Test® [95], and formol-ether concentration techniques [96]. In these methods, several technical factors can alter the sensitivity of strongyloidiasis diagnosis, for example, sample homogenization, stool processing delays, erroneous morphological differentiations from nematode larvae, and inadequate numbers or preservation of samples [97]. The Baermann-Moraes is inexpensive and simple to perform and one of the most used parasitological methods for nematode larva identification in routine laboratories; however, it is less sensitive than the CPA technique [98–100]. Additionally, CPA allow the differential diagnosis between species of* S. stercoralis* and hookworms based on the type of migration paths (furrows) left by the larvae in agar plates [100].

Due to the limitations of parasitological methods, the use of more sensitive immunological tests is critical for the diagnosis of strongyloidiasis. ELISAs are the most widely used immunodiagnostic methods and have shown sensitivities and specificities above 70.0%, depending on the screened antigen and antibody isotype [101–104]. However, because of difficulties in the production and the standardization of antigens capable of providing reproducible results, the use of ELISAs remains limited [30]. Uncertainties regarding positive reactions may occur in cases of hidden strongyloidiasis (i.e., low parasite burdens), immunological memory of past infections, or the presence of similar antigens among helminths [104]. In fact, the presence of cross-reactivity with other helminths is considered to be one of the most important limitations in strongyloidiasis immunoassays [101, 105], especially in endemic countries. Conversely, in areas where the infection is uncommon, the detection of specific antibodies is more reliable.

In a recent study, the diagnosis of strongyloidiasis by IgE-ELISA, using as antigen the “strongylastacin,” an enzyme secreted by infective larvae of* S. stercoralis*, showed high sensitivity and high specificity [106]. The detection of IgA anti-*S. stercoralis* can also aid in the diagnosis of strongyloidiasis [107], especially in patients without excretion of rhabditoid larvae in feces. Due to the immunomodulation of IgA during infections, the results of stool tests are usually negative [63]. The suggested mechanisms by which IgA modulates the removal of host larvae include reductions of female fertility and of the intensity of the autoinfection [108]. ELISAs can also be applied for the detection of* S. stercoralis* antigens in stool samples (coproantigen), even though these tests have not yet been standardized [109, 110].

Other immunoassays, such as western blots, can be used to confirm strongyloidiasis diagnoses in cases of discordant serological tests or negative parasitological samples. Silva et al. [105] demonstrated that 96.0% of sera from patients with strongyloidiasis recognized immunodominant antigens of* Strongyloides ratti*. According to western blots, the sera from infected patients can recognize different molecular antigenic patterns. For instance, Sato et al. [111] revealed four antigens with molecular weights of 97, 66, 41, and 26 kDa; Ravi et al. [112] described a 38 kDa molecule and Sudré et al. [113] identified 26 and 33 kDa immunodominant molecules. However, no consensus or agreement in the literature has been reached regarding immunodominant* S. stercoralis* molecular patterns that can be used as standardized references for the diagnosis of strongyloidiasis.

Polymerase chain reaction (PCR) assays are also considered to be sensitive and specific method for the diagnosis of* S. stercoralis* [114, 115]. However, as in the parasitological analysis of feces, PCR assays depend on the shedding of larvae, which only occurs on an intermittent basis. Moreover, PCR assays are expensive and require skilled labor and complex infrastructure not commonly used in routine clinical laboratories, especially in those of endemic areas.

Eosinophilia has also been considered as a nonspecific laboratory marker for the screening of chronic strongyloidiasis, especially for asymptomatic individuals from endemic areas [27, 116, 117]. The location of parthenogenetic females in intestinal mucosa and larvae transit through tissues, especially the lungs, stimulate immune responses mediated by eosinophils [2, 56].

Despite the several existing strongyloidiasis diagnosis methods, there remains no ideal standard tool. A reliable diagnosis is particularly important for patients from endemic areas, especially those in groups at higher risk of developing hyperinfection or disseminated strongyloidiasis.

Due to the autoinfection process, all patients should be treated to prevent the development of strongyloidiasis severe cases. Albendazole, thiabendazole, and ivermectin have been used as therapeutic agents for strongyloidiasis. For many years, thiabendazole was the treatment of choice. However, the use of the drug was associated with unpleasant side effects, such as nausea, vomiting, malaise, and neuropsychiatric symptoms, and is no longer available in some countries [118–120]. Albendazole, another benzimidazole compound with board-spectrum anthelmintic activity, remains widely used for strongyloidiasis treatment in some countries where oral ivermectin is not yet available [119, 121]. However, the efficacy of albendazole in* S. stercoralis* treatment is inconsistent, especially for hyperinfection risk groups [8, 122–124].

Currently, ivermectin seems to provide the best outcomes for treating* S. stercoralis* and is strongly recommended for severe cases, such as hyperinfection and disseminated disease. Ivermectin—a member of a family of macrolytic lactones, the avermectins—has broad spectrum activity against parasites. It binds to glutamate-gated chloride ion channels, which are present in invertebrate nerve and muscle cells, and causes paralysis and death of the parasite. It does not easily cross the blood-brain barrier in humans and has a low affinity for mammalian ligand-gated chloride channels [125]. Ivermectin demonstrated better efficacy than albendazole and had less adverse effects than thiabendazole. Moreover, adverse reactions after ivermectin treatment are rarely experienced, transient, and well tolerated [119, 122, 126].

The administration of two single doses of 200 *μ*g/kg ivermectin given two weeks apart is more suitable for treating chronic strongyloidiasis than a single dose [120, 127]. In immunocompromised patients, to prevent the recurrence of hyperinfection syndrome, two doses of ivermectin are administered every two weeks, for six weeks [128]. Thereafter, patients with hyperinfection require follow-up stool exams. These exams use highly sensitive diagnostic methods and are performed for 2–4 weeks to confirm the clearance of the infection because the internal parasite development cycle is a two-week long process [128–130]. In patients presenting with stool examinations positive for* Strongyloides* or persistent symptoms, if a recrudescence of larvae is observed, retreatment is indicated.

## 6. Conclusion

Chronic alcoholism can cause damage to a patient, including adverse effects in the gastrointestinal tract and the immune system. These effects may render patients more susceptible to parasitic infections. Studies have demonstrated a higher frequency of* Strongyloides stercoralis* in chronic alcoholics when compared with nonalcoholics. This phenomenon has been attributed to the breakdown of local protective barriers, higher exposure to pathogens, malnutrition, endogenous production of corticosteroids, and alterations to host immune defense mechanisms.

Although many hypotheses have been raised, due to the multifactorial pathogenic effects induced by alcohol, the specific mechanism or group of mechanisms involved in the high susceptibility of alcoholics to* S. stercoralis* infection and hyperinfection are not completely understood. Further studies with large numbers of alcoholic patients, infected and noninfected with* S. stercoralis*, should be conducted. These studies should focus on cortisol levels, local and systemic immune responses to parasites, mucosa alterations, and estimations of parasite loads.

In high risk groups, such as patients with chronic alcoholism, the diagnosis of strongyloidiasis should be performed using more sensitive parasitological methods, such as with agar plate cultures, combined with an immunological test for the detection of anti-*S. stercoralis* antibodies. The recommended treatment is ivermectin, even in the absence of symptoms, due to possibility of autoinfection and the development of severe cases of hidden strongyloidiasis.

## Additional Points

Chronic alcoholism is a risk factor that leads to strongyloidiasis infection and hyperinfection. Regular ethanol intake may lead to an immune modulation or alteration in corticosteroid metabolism, favoring* S. stercoralis* autoinfection. Both chronic ethanol addiction and* S. stercoralis* hyperinfection can significantly damage the intestinal mucosa. The shedding of large numbers of larva may synergistically act with alcohol in eroding the mucosal layer and cause blood loss in the intestine, leading to malabsorption. Early diagnoses and treatments of strongyloidiasis in alcoholic patients are essential in endemic areas to prevent hyperinfection and the dissemination of the parasite.
